# Molecular Dynamic Simulation to Explore the Molecular Basis of Btk-PH Domain Interaction with Ins(1,3,4,5)P4

**DOI:** 10.1155/2013/580456

**Published:** 2013-11-06

**Authors:** Dan Lu, Junfeng Jiang, Zhongjie Liang, Maomin Sun, Cheng Luo, Bairong Shen, Guang Hu

**Affiliations:** ^1^Center for Systems Biology, Soochow University, Suzhou 215006, China; ^2^Drug Discovery and Design Center, State Key Laboratory of Drug Research, Shanghai Institute of Materia Medica, Chinese Academy of Sciences, Shanghai 201203, China; ^3^Department of Bioinformatics, Medical College, Soochow University, Suzhou 215123, China

## Abstract

Bruton's tyrosine kinase contains a pleckstrin homology domain, and it specifically binds inositol 1,3,4,5-tetrakisphosphate (Ins(1,3,4,5)P4), which is involved in the maturation of B cells. In this paper, we studied 12 systems including the wild type and 11 mutants, K12R, S14F, K19E, R28C/H, E41K, L11P, F25S, Y40N, and K12R-R28C/H, to investigate any change in the ligand binding site of each mutant. Molecular dynamics simulations combined with the method of molecular mechanics/Poisson-Boltzmann solvent-accessible surface area have been applied to the twelve systems, and reasonable mutant structures and their binding free energies have been obtained as criteria in the final classification. As a result, five structures, K12R, K19E, R28C/H, and E41K mutants, were classified as “functional mutations,” whereas L11P, S14F, F25S, and Y40N were grouped into “folding mutations.” This rigorous study of the binding affinity of each of the mutants and their classification provides some new insights into the biological function of the Btk-PH domain and related mutation-causing diseases.

## 1. Introduction

Bruton's tyrosine kinase (Btk) is a member of the Tec family of kinases and the only known one associated with human disease [1, 2]. Previous studies have indicated the significance of Btk in B-cell development, differentiation, and signaling [3, 4]. Once the Btk-dependent signal transduction pathway is inactivated, B cells remain at the pre-B-cells stage leading to X-linked agammaglobulinemia (XLA) in humans, which is one of the most frequently inherited immunodeficient disorders in human, and X-linked immunodeficiency (Xid) in mice [5–8].

The Btk protein contains Src-homology 2 and 3 domains (SH2 and SH3), a catalytic SH1 domain, a Tec-homology (TH) domain, and an N-terminal pleckstrin homology (PH) domain [9–11]. Studies have shown that XLA mutations in Btk can be mapped to all five domains of the kinase, which are critical for signal transmission. Several missense mutations in the PH domain have been widely studied and so far considered to be the only known cause of the disease [12]. The PH domain is responsible for binding with phosphatidylinositols, showing the importance for the regulation of membrane targeting. Thus, a mutation in the PH domain can influence the binding affinity with a ligand, membrane targeting, and the activation of Btk [13, 14]. Of the various phosphatidylinositols, the Btk-PH domain has higher specificity and binding affinity with inositol 1,3,4,5-tetrakisphosphate (Ins(1,3,4,5)P4) [15–17]. The crystal structure of a complex of the Btk-PH domain with Ins(1,3,4,5)P4 (PDB ID code: 2Z0P [18]) shows that the Btk-PH domain recognizes Ins(1,3,4,5)P4 in a canonical manner [19, 20].

The PH domain is a structural protein domain containing approximately 120 amino acid residues that retains a highly conserved three-dimensional organization of different proteins, despite their poorly conserved primary sequences [21–23]. The core structure is a **β**-sandwich of two almost orthogonal **β**-sheets consisting of three and four strands, respectively. The opposite edge of the structure is capped by an amphipathic C-terminal **α**-helix. Six loops connect the **β**-strands, while the amino acids in the **β**1*-*β**2 and **β**3*-*β**4 loops form a hydrogen bond network that interacts with Ins(1,3,4,5)P4 [19].

According to previous studies, XLA-causing mutations can be mainly classified into two groups [24, 25]. The first group of mutations appears to prevent the formation of stable native-like structures, which are identified as “folding mutations.” In the second group named as “functional mutations,” the mutations do not affect the overall fold, though they may disrupt the ligand binding affinity leading to functional defects. Specially, K12 and R28 are significant in most functional mutations involved in the interaction with a ligand because a functionally based covariant pair is created, which contacts with a bound negatively charged ligand [26]. Generally, experimental studies have indicated the biological influence of amino acid mutation [24, 25, 27], but the proposed explanations do not exactly reveal much detailed molecular basis of the effects of amino acid mutations, such as a change in hydrogen bonds, binding sites, and binding free energy.

In this paper, we have studied Btk-PH domain binding with the Ins(1,3,4,5)P4 structure in wild type and 11 well-known mutants [24, 25, 27], L11P, K12R, S14F, K19E, F25S, R28C/H, Y40N, E41K, and K12R-R28C/H. The method of molecular mechanics/Poisson-Boltzmann solvent-accessible surface area (MM/PBSA) was used to compare the binding affinity [28–32]. Three main aims were mainly raised and discussed in this study: (1) the classification of the eleven mutants, (2) the effects of amino acid mutations on the biological function of the Btk-PH domain, and (3) the possible coordinate interactions of amino acid residues. Our simulations indicated that mutations K12R, K19E, R28C/H, E41K, and K12R-R28C/H were “functional mutations,” because negative binding free energies were identified in the MM/PBSA calculation for these structures, whereas the others were considered to be “folding mutations” whose binding free energies are positive. The ligand clearly moves away from the binding pocket in R28C/H mutation structures. In K12R mutation structures, the ligand sites in the top of the binding Pocket, which results in the evident decrease of binding free energy. Thus, the biological function of Btk is weakened in all three mutant structures. The K19E mutation leads to a major change in the **β**1-**β**2 loop, leading to weak membrane targeting and the suppression of Btk activation. The E41K mutation increases the positive charge of the **β**3-strand, which increases the positively charged surface. This may provide a cavity for binding with another ligand, which could enhance the membrane targeting and activation of Btk. Remarkably, the two double amino acids mutant structures K12R-R28C/H retain a similar ligand binding affinity to the wild type structure, because new coordinate interactions by the amino acid pairs R12 and K53 are found to play important roles in the binding with Ins(1,3,4,5)P4. We hope that our findings help to explain the relationship between residue mutations and biological function, as well as the molecular basis of related diseases.

## 2. Materials and Methods

### 2.1. Btk PH-Ins(1,3,4,5)P4 and Mutation Structures

The X-ray structure of the PH domain of Btk (PDB ID code: 2Z0P (A chain); resolution: 2.58 Å) [18], which is bound to Ins(1,3,4,5)P4, was used as the initial structure for all simulations. The PH domain has the common PH domain fold, which has a seven-stranded antiparallel *β*-sheet grouped into a *β*-sandwich and a *α*1 helix. For far distance away from the binding pocket, the Zn^2+^ ion in the structure was deleted for simplification and the forces between the Zn^2+^ ion and original four amino acids, His143 and Cys154/155/165, were manually added to reduce the flexibility of protein structure. Mutant structures (L11P, K12R, S14F, K19E, F25S, R28C/H, Y40N, E41K, and K12R-R28C/H) were obtained and visualized using the Sybyl software package (Tripos, St. Louis, MO). Ligand of Ins(1,3,4,5)P4 was extracted from the PH domain complex as an independent small molecule for the molecular dynamics (MD) and MM/PBSA simulation. All initial mutation structures were minimized by Sybyl using a distance-dependent dielectric function, with a nonbonded cut-off of 8 Å. Amber charges were assigned to the protein while Gasteiger-Hückel charges were given to Ins(1,3,4,5)P4. The whole system was minimized until no more atom collisions were found. 

### 2.2. MD Simulation

Before the MD simulation, the charge information of Ins(1,3,4,5)P4 was calculated using the RESP method [33] encoded in the AMBER suite program (version 9) [34], followed by Gaussian 03 for calculations at the Hartree-Fock (HF)/6-31G* level. After the application of the AMBER03 force field to proteins [35] and the general AMBER force field (gaff) to ligands [36], each complex was loaded into the AMBER9 program, adding Na^+^ ions with a 1 Å grid to neutralize the system. Finally, an 8 Å water TIP3PBOX was loaded into the system to form a complex environment. Subsequently, energy minimization was performed to remove any inappropriate contacts.

MD simulations were conducted with a nonbonded cut-off of 8 Å, the dielectric constant of 1.0, integration step of a 2 fs time interval, and Particle Mesh Ewald (PEM) for long-range electrostatic interaction calculation [37], using the following protocol: (1) 500 ps of heating equilibration with weak restraints on each complex in Cartesian space using a harmonic potential, where the temperature was gradually increased to 300 K, with constant volume, and Langevin dynamics for temperature control; (2) 500 ps of density equilibration, 300 K thermal bath at 1.0 atm of pressure (atm = 101.3 kPa) periodic boundary conditions with isotropic position scaling, and Langevin dynamics for temperature control; (3) 1 ns of constant pressure equilibration with weak restraint on the ligand and 20 ns of constant pressure equilibration at 300 K.

### 2.3. MM-PBSA Calculations for Binding Free Energy

Binding free energy (Δ*G*_bind_) of ligand on Btk-PH domain was calculated by single trajectory method of MM/PBSA, according to the following equation:

(1) $$\begin{array}{c} \Delta G_{\text{bind}}=G^{\text{complex}}-G^{\text{PH}}-G^{\text{li}}, \end{array}$$

where the free energies of complex, PH domain, and ligand are denoted as *G* ^complex^, *G* ^PH^, and *G* ^li^, respectively. Free energy (*G*) was calculated according to following equations:

(2) $$\begin{array}{c} G=E_{\text{gas}}+G_{\text{sol}}-TS_{\text{config}},\\ E_{\text{gas}}=E_{\text{vdw}}+E_{\text{ele}}+E_{\mathrm{int}},\\ G_{\text{sol}}=G_{\text{elec}}+G_{\text{SA}}, \end{array}$$

where *E*_gas_ defines the molecular mechanical energy, *G*_sol_ defines a solvation free energy, which is further decomposed to polar (*G*_elec_) and nonpolar (*G*_SA_) terms, and *S*_config_ defined as configurational entropy, which is normally derived from normal model analysis. *E*_gas_ was the sum of contributions from internal energies including bond, angle, and torsion angle energies (*E*_int⁡_), electrostatic energy (*E*_ele_), and van der Waals energy (*E*_vdw_), which were calculated using the same force field as that of MD simulations with no cut-off. Notably, the *E*_int⁡_ was assigned to zero in (1), since the torsion angle energies of the complex and the separated part were calculated from the same trajectory. In the MM/PBSA calculation, 2000 snapshots of each model were extracted from the last 10 ns of the trajectories at time intervals of 5 ps.

## 3. Results and Discussions

**Figure 1:**
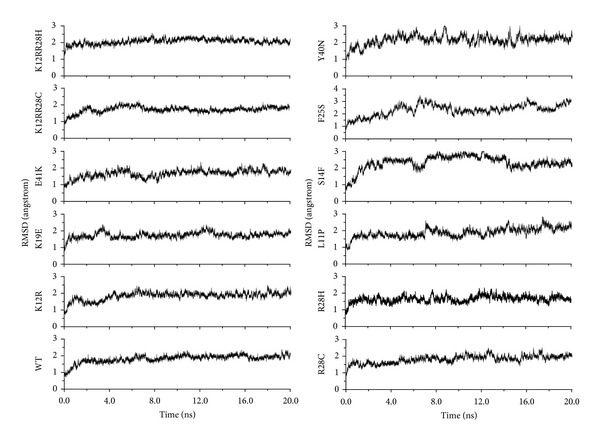
Time dependence of the root-mean-square deviations (RMSDs) for the C^*α*^ atoms from their initial structure of 20 ns MD simulations of all complex structures.

**Figure 2:**
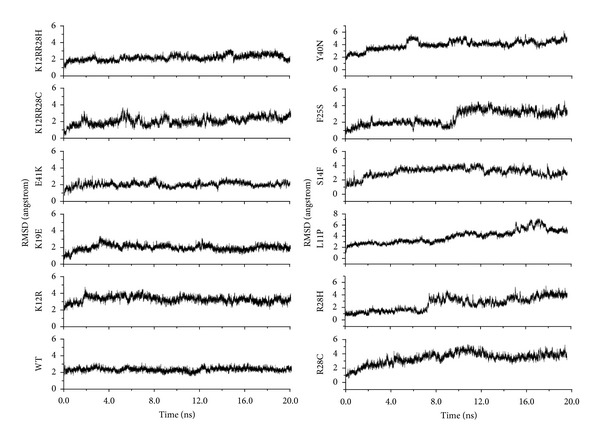
Time dependence of the root-mean-square deviations (RMSDs) for the ligand from its initial structure of 20 ns MD simulations of all complex structures.

MD simulations were used to investigate the complexes of 11 mutants and wild type PH domain and the interaction behavior between the PH domain and the ligand Ins(1,3,4,5)P4. Details of the simulation setup are provided in Materials and Methods. The root-mean-square deviation (RMSD) values of the C^*α*^ atoms relative to the initial coordinates were calculated using the 20 ns trajectory data. As shown in Figure 1, the RMSD tended to be flat after 5 ns' simulation in WT, K12R, K19E, E41K, K12R-R28C, K12R-R28H, R28C, and R28H at about 2 Å, indicating stable conformations. As for the RMSD values for other four mutants, relative large fluctuations were observed during the 20 ns' simulation, suggesting unstable structures and maybe poor binding affinities between the protein and the ligand. Meanwhile, the RMSD value of the ligand Ins(1,3,4,5)P4 was also calculated to further indicate the stability of the structures. In Figure 2, it was obvious that the ligand of WT, K12R, K19E, E41K, K12R-R28C, and K12R-R28H was more stable than that of R28C, R28H, L11P, S14F, F25S, and Y40N. Therefore, it was believed that R28C, R28H, L11P, S14F, F25S, and Y40N mutants might share some similar instabilities, which was in good accordance with the predefined classifications based on experimental results [27]. 

In order to offer a better criterion for classification of the mutants, the MM/PBSA method was used to estimate the binding affinities of the PH domain and the ligand in the 12 structures. Energy results for all the MM/PBSA calculations shown in Table 1 indicated that these mutations could be clearly divided into two groups. It was convinced that the “folding mutations” could not fold into a stable native-like structure to perform the function of the PH domain [27]. In our results, it is obvious that the four mutations, L11P, S14F, F25S, and Y40N, own a positive binding free energy, though the enthalpy change is favorable, implying the formation of complex is not allowed by thermodynamics. As aforementioned, these four mutations also had unstable RMSD value. Therefore, it is most likely that the four “folding mutations” will result in the loss of function of the PH domain completely, although S14F was considered to be a “functional mutation” in an earlier study [24, 25]. On the contrary, the other 7 mutations have negative binding free energies. With the stable RMSD values, it is proposed that the mutations of K12R, K19E, R28C/H, E41K, and K12R-R28C/H may be considered as “functional mutations,” which do not affect the overall fold but can be expected to disrupt the binding affinities between the protein and the ligand [24, 27]. In this paper, the binding free energies of seven mutation structures were carefully compared to the wild type, using Δ*G*_bind_ as the criterion. Different mutational simulations provided important information about the affinity drift of the ligand-binding protein complex, which were further discussed below.

### 3.1. Structural and Binding Affinity Analysis of “Functional Mutations”

According to the MM/PBSA simulations, the binding affinity of the 7 “functional mutations” was ranked to offer a better understanding of risks in disease, which was displayed in Table 1. 

**Figure 3:**
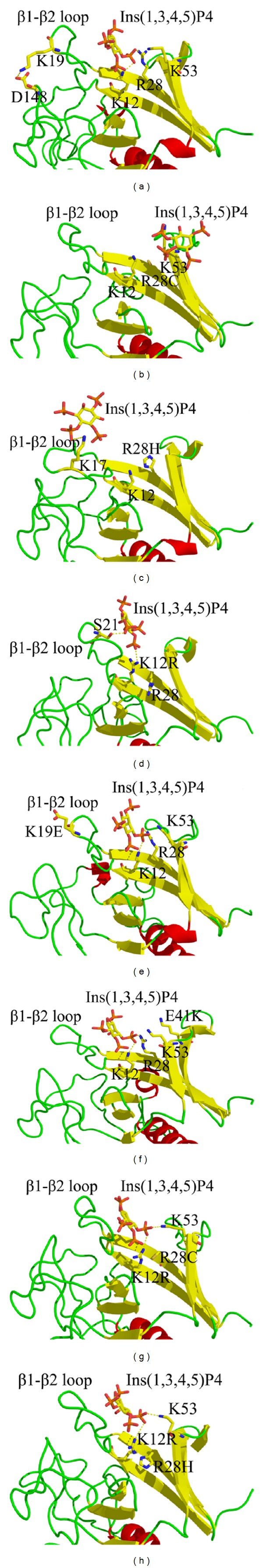
The snapshot structures taken at time 20 ns of wild type and all “functional mutations” complexes from MD simulation. Ligands and surrounding important residues are labeled and shown in stick, while hydrogen bonds are displayed in yellow dashed lines. (a) Wild type complex; (b) R28C mutation complex; (c) R28H mutation complex; (d) K12R mutation complex; (e) K19E mutation complex; (f) E41K mutation complex; (g) K12R-R28C mutation complex; (h) K12R-R28H mutation complex.

As aforementioned, the RMSD values of the ligand in R28C and R28H mutants were greater than 4, which was two times larger than that of wild type. Therefore, it is most likely that the ligand completely moves away from the original binding pocket of the PH domain and this was consistent with the result of the binding free energy calculation for R28C/H mutants (Table 1). It is clear that the neutral charged cysteine has a sulfur atom in its side chain, which imposes a larger steric restriction compared with arginine. Although it has the same positive charge as arginine, histidine has a five-member ring that produces more steric hindrance than the side chain of arginine. Structural analysis in Figure 3 verified that the ligand was already out of the binding pocket. The mutations removed the hydrogen bond interaction between R28 and the ligand while forming new hydrogen bond interactions with residues on the *β*4 strand (K53) and *β*1-*β*2 loop (K17), respectively, which would not provide suitable side chains to bind with the ligand. Accordingly, it was considered that the R28C/H mutations had greatly reduced the binding affinity with the ligand. And the biological function of the two mutants was severely weakened, which may be considered as a primary factor that leads to associated diseases such as XLA and Xid [5].

As shown in Table 1, according to the MM/PBSA simulations, the free binding free energies of the mutants K19E, E41K, and K12R-R28C/H were similar to the wild type, whereas the K12R mutant was relatively smaller (−14.08 kcal/mol). Furthermore, compared with the wild type, the ligand was still located in the binding pocket of these mutants, as shown in Figures 3(d)–3(h), although there were different ligand-conformational changes due to hydrogen bond rupture. The time evolution of the root-mean-square fluctuation (RMSF) from the initial structure was also analyzed to further evaluate the ligand binding behavior of these mutations.

Structural analysis of the wild type showed that K12, which was located at the bottom of the binding pocket, formed hydrogen bonds with the 3- and 4-phosphates of the ligand, which is critical for the twist of *β*1-*β*2 loops [24]. In the K12R mutant, there was obvious decrease in binding free energy in the MM/PBSA simulation (Table 1), which could be explained by the difference in the conformation and the change in the hydrogen bond interactions of the ligand. Sharing the same positive change as lysine, the three atoms in arginine tail formed a conjugated system, which decreased the flexibility and increased the steric hindrance of this residue, leading to the conformational change required for ligand binding. The hydrogen bond with the 3-phosphates of the ligand was also fully ruptured in K12R mutant, resulting in a loss of specificity in the Btk-PH domain for the Ins(1,3,4,5)P4 ligand. As shown in Table 2, the K12R mutant abolished the hydrogen bond network formed by K12, R28, and the ligand; thereby the contribution of the conserved covariation pair on the biological function of the Btk-PH domain was removed.

**Figure 4:**
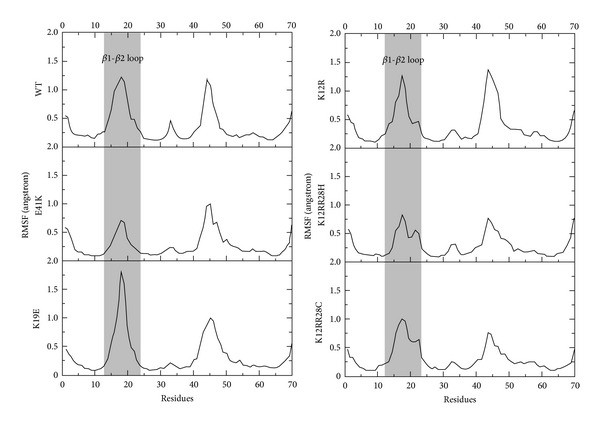
Residue fluctuations obtained by average residual fluctuations between residues 1 and 70 for six simulations, wild type (WT), K19E, E41K, K12R, and K12R-R28C/H, over the 20 ns simulations are illustrated in solid lines. The residues of **β**1-**β**2 loop are highlighted.

In the wild type, as shown in Figure 3(a), K19 contacts D148 via a hydrogen bond, which moves the *β*1-*β*2 loop closer to the tail of the structure and moves it away from the ligand. In the mutant K19E, a stable hydrogen bond network is formed among S14, T20, and the ligand after the rupture of the hydrogen bond between E19 and D148 (Table 2), which leads to a higher binding free energy with the ligand than the wild type while leading to an almost 90° change in the direction of the *β*1-*β*2 loop, as shown in Figure 3(e). The RMSF analysis in Figure 4 also confirmed that the *β*1-*β*2 loop had much greater fluctuations than the wild type. Therefore, it is possible the significant change in the *β*1-*β*2 loop of this mutation could eliminate the positively charged surface that interacts with the negatively charged membrane surface, resulting in poor membrane targeting and incomplete activation of Btk. This may mean that the biological function of Btk is not fully available. Therefore, K19E was also a loss-of-function mutation, although it had a similar binding affinity with the ligand than the wild type, which was in good agreement with the experimental results [24, 27].

Compared with K19E, the mutant of E41K has the opposite effect on the biological function of Btk. As shown in Figure 3(a), E41 was located in the *β*3 strand with some distance from the ligand, but it is known to have great importance in the contact with the membrane. Despite the higher steric hindrance after the substitution of glutamate with lysine, the ligand slightly shifts to the *β*1-*β*2 loop in Figure 3(f), which results in a reduced flexibility of the *β*1-*β*2 loop (RMSF in Figure 4). The binding free energy of the E41K mutant was also smaller than the wild type, as shown in Table 1. Thus, E41K may retain most of the biological functionality of the wild type. However, the mutation in E41K increased the positively charged complex surface of the region located above the *β*3 and *β*4 strand, which was favorable for interaction with the negatively charged membrane surface, while it also offered a second ligand binding site. Therefore, it is likely that the E41K mutant may increase the activation of Btk, which is known as a gain of function [24, 27].

### 3.2. Covariant Pair Identification in K12R-R28C/H Mutations

Of the disease-causing mutations, the mutations in K12 and R28 always coexist with other mutations, although with lower frequencies, which may be due to the diversity of the PH domain. Single mutations of K12 and R28 have been known to weaken the biological function of the Btk-PH domain to different degrees, while it is also reported that K12 and R28 can form a conserved covariant pair that maintains the binding affinity of Ins(1,3,4,5)P4, which plays an important role in the biological function of the Btk-PH domain [26]. Therefore, the double residue mutations of K12R-R28C/H were closely analyzed to underly the molecular basis. Compared with the wild type structure, the ligand was still located in the bottom of the binding pocket of the two mutations (Figures 3(g) and 3(h)). The MM/PBSA simulations indicated a similar free binding energy for K12R-R28H and K12R-R28C (Table 1). This could be explained by the formation of a new covariant pair between R12 and K53 as K12-R28. Unlike the K12R mutation, the hydrogen bond network of 3-phosphates was kept through R12 and K53 in the mutation of K12R-R28C/H, which ensured the preservation of ligand specificity, whereas all hydrogen bond interactions between H28/C28 and ligand were ruptured (Table 2). Interestingly, a new conserved hydrogen bond was discovered between K53 and the ligand during the MD simulations. Instead of the covariant pair K12-R28, we believe that R12-K53 formed a similar odd positively charged pair that made contact with a bound negatively charged inositol phosphate and thereby can maintain the biological function of the K12R-R28C/H mutants.

## 4. Conclusions 

Twelve complex systems, including eleven mutants and one wild type PH domain, have been comprehensively studied by integrating MD and MM/PBSA simulations, to reveal the detailed molecular basis. As a result, L11P, S14F, F25S, and Y40N were considered to be “folding mutations,” whereas mutants K12R, K19E, R28C/H, E41K, and K12R-R28C/H were classified into “functional mutations.” The effects of “functional mutations” on the biological function of the Btk-PH domain have also been predicted. In the R28C/H mutation, the ligands completely left the binding pocket and formed new interactions with the *β*1-*β*2 loop and the *β*4 strand, respectively. For the K12R mutation, the biological function of the Btk-PH domain may be reduced by the lack of hydrogen bond interaction with 3-phosphates when the ligand is formed, which determines the specificity of the Btk-PH domain. As for the K19E mutation, the change in the residue charge and the trend of the *β*1-*β*2 loop eliminated the positively charged surface that interacted with the negatively charged membrane surface, resulting in poor membrane targeting and incomplete activation of Btk. In contrast, E41K increased the positive surface of the Btk-PH domain, thereby increasing the possibility of the binding with a second ligand, which may result in a gain of function. Of note, the K12R-R28C/H mutant retained a high binding affinity without the displacement of the ligand from the binding pocket. A new odd positively charged pair was formed between R12 and K53 in the K12R-R28C/H mutation, which was similar to the K12-R28 in the wild type. This formation may interact with a bound negatively charged inositol phosphate, which may preserve the biological function of the Btk-PH domain and this should be further investigated experimentally.

In summary, our MD and MM/PBSA simulations of the twelve structures may provide some new insights into PH domain and their associated diseases. Based on the binding free energy, binding site of the ligand, and hydrogen bond interactions, we have predicted the effects of mutations on the biological function of the Btk-PH domain.

## Figures and Tables

**Table 1:** Ranked calculated binding free energies (kcal/mol) using MM/PBSA for the twelve protein-ligand systems.

	ELE	VDW	PB	SA	PBTOT	*T*Δ*S*	Δ*G*_bind_
WT	−3031.77 (9.01^a^)	−0.16 (0.07)	2961.60 (7.63)	−4.28 (0.47)	−74.61 (5.10)	−45.29 (1.43)	−29.32 (1.49)
K12R-R28C	−3119.66 (10.33)	−0.12 (0.05)	3044.04 (8.61)	−4.25 (0.18)	−79.99 (3.37)	−52.50 (1.45)	−27.49 (1.06)
K19E	−3143.08 (8.92)	−0.17 (0.03)	3069.08 (7.38)	−4.21 (0.16)	−78.38 (5.33)	−51.09 (1.60)	−27.29 (0.73)
K12R-R28H	−3029.46 (8.74)	−0.05 (0.02)	2962.73 (6.43)	−4.12 (0.21)	−73.30 (2.02)	−51.66 (1.37)	−21.64 (0.66)
E41K	−2855.47 (7.73)	−0.28 (0.16)	2789.39 (5.95)	−4.16 (0.23)	−70.52 (3.12)	−50.08 (1.52)	−20.44 (0.38)
K12R	−2922.34 (9.17)	−0.08 (0.03)	2859.80 (8.38)	−4.30 (0.21)	−66.92 (3.62)	−52.84 (1.57)	−14.08 (0.94)
R28C	−2612.17 (5.95)	−1.90 (0.23)	2552.82 (5.03)	−3.69 (0.16)	−64.94 (1.87)	−57.28 (2.47)	−7.66 (0.03)
R28H	−2591.08 (6.16)	−1.61 (0.17)	2533.38 (4.65)	−3.70 (0.17)	−63.01 (1.16)	−56.80 (2.54)	−6.21 (0.02)
S14F	−2573.79 (7.06)	−3.37 (0.74)	2545.38 (6.99)	−3.42 (0.25)	−35.20 (2.47)	−57.49 (3.02)	22.29 (0.98)
L11P	−2599.24 (7.61)	−1.75 (0.41)	2578.10 (6.70)	−3.94 (0.33)	−26.83 (1.42)	−58.75 (4.47)	31.92 (2.02)
Y40N	−2550.35 (7.0)	−2.57 (0.59)	2531.48 (6.23)	−3.92 (0.27)	−25.36 (1.84)	−58.34 (3.89)	32.98 (1.68)
F25S	−2501.74 (7.17)	−2.59 (0.69)	2487.39 (6.64)	−3.77 (0.19)	−20.71 (1.54)	−58.32 (4.15)	37.61 (1.82)

^a^The statistical error was estimated on the basis of the deviation between block averages.

ELE: electrostatic energy; VDW: van der Waals energy; PB: the PB solvation energy; SA: the surface area energy (nonpolar solvation energy); PBTOT: sum of ELE, VDW, PB, and SA.

The absolute temperature (*T*) was set to 300 K in the MM-PBSA calculations. Δ*G*_bind_: subtracting *T*Δ*S* from PBTOT.

**Table 2:** Comparison of hydrogen bond interactions of five “functional mutations” and wild-type (WT) structures. Only hydrogen bonds with occupation ratio of the trajectory greater than 30%, atomic distance of two heavy atoms smaller than 3 Å, and atomic angle smaller than 60° are presented. The rank of amino acid according to its occupation ratio of the trajectory is from large to small.

WT	E41k	K19E	K12R	K12R-R28H	K12R-R28C
Arg28 3HB with 3-PG^a^ Lys12 3HB with 3-PG Ser14 1HB with 4-PG Lys26 4HB with 1-PG Ser 21 2HB with 5-PG 1HB with 6-OH^*β*^ Gln15 1HB with 4-PG Lys17 2HB with 4-PG Lys53 2HB with 3-GP	Arg28 3HB with 3-PG Lys12 3HB with 3-PG Ser 14 2HB with 4-PG Ser 21 2HB with 5-PG Gln15 1HB with 4-PG Lys26 2HB with 1-PG Lys53 2HB with 3-GP	Arg28 3HB with 3-PG Lys12 3HB with 3-PG Ser 14 1HB with 4-PG Thr 20 1HB with 5-PG Lys17 2HB with 4-PG Gln15 1HB with 4-PG Ser 21 2HB with 5-PG 1HB with 6-OH Lys26 2HB with 1-PG Lys53 2HB with 3-PG	Arg12 2HB with 4-PG Ser 21 1HB with 5-PG Lys17 2HB with 5-PG Ser 14 1HB with 4-PG Gln15 1HB with 4-PG Lys26 1HB with 1-PG	Arg12 3HB with 3-PG Ser 14 1HB with 4-PG Lys53 2HB with 3-PG Gln15 3HB with 4-PG Lys17 2HB with 4-PG Ser 21 1HB with 5-PG 1HB with 6-OH Lys26 1HB with 1-PG	Arg12 3HB with 3-PG Lys53 2HB with 3-GP Ser 14 1HB with 4-PG Gln15 3HB with 4-GP Lys17 2HB with 4-PG Ser 21 2HB with 5-PG 1HB with 6-OH Lys26 1HB with 1-PG

^a^HB and PG represent hydrogen bond and phosphate group, respectively.

^*β*^OH represents OH group in the inositol ring.
